# Leveraging expression quantitative trait loci information in single-cell resolution to identify cell-specific genes for gestational diabetes

**DOI:** 10.1371/journal.pone.0355339

**Published:** 2026-07-30

**Authors:** Wenyan Zhu, Xiaowei Wu, Rongkui Hu

**Affiliations:** 1 Department of Epidemiology, Center for Global Health, School of Public Health, National Vaccine Innovation Platform, Nanjing Medical University, Nanjing, Jiangsu, China; 2 Department of Breast Surgery, The First Affiliated Hospital of Nanjing Medical University, Nanjing, Jiangsu, China; 3 Gynecology Department, Affiliated Hospital of Nanjing University of Chinese Medicine, Jiangsu Province Hospital of Chinese Medicine, Nanjing, Jiangsu, China; Shanghai Jiao Tong University, CHINA

## Abstract

**Background:**

Gestational diabetes mellitus (GDM) is a common pregnancy complication with long-term metabolic consequences for both mother and offspring. While genome-wide association studies (GWAS) have identified risk loci, the cell-type-specific genetic architecture remains poorly characterized due to the limitations of bulk-tissue analyses.

**Methods:**

We integrated GWAS summary statistics from FinnGen (12,332 cases, 131,109 controls) with single-cell expression quantitative trait loci (sc-eQTL) data from 12 immune cell types in the OneK1K cohort. Using the OTTERS framework and ACAT-O omnibus testing, we performed single-cell transcriptome-wide association studies (scTWAS) to identify GDM-associated genes. We performed bulk-level TWAS as a sensitivity analysis. To investigate the function of significant genes, we performed functional enrichment.

**Results:**

We detected 14 unique genes significantly associated with GDM across immune cell types (FDR < 0.05), with the strongest signals in CD4^+^ T cells and monocytes. Notably, *ERAP1* and *ERAP2* showed associations in 10 of 12 cell types (ACAT. *P* = 2.17 × 10^−5^), and *RIOK2* emerged as a shared regulator. Gene Ontology analysis consistently highlighted “antigen processing and presentation via MHC class I” as the top enriched pathway (FDR < 10^−5^). In contrast, traditional bulk TWAS using GTEx whole blood identified only two genes with nominal associations.

**Conclusion:**

Our sc-TWAS identifies cell-type-specific GDM-associated genes that are not detected in bulk tissue, highlighting dysregulated antigen presentation in peripheral immune cells. Colocalization prioritizes monocytic *LNPEP* as the primary causal candidate, while associations for *ERAP1*, *ERAP2*, and *RIOK2* likely reflect complex LD or tissue-specific effects.

## Introduction

Gestational diabetes mellitus (GDM) is a type of diabetes that is diagnosed during the second or third trimester of pregnancy and was not clearly overt diabetes before pregnancy [1]. It develops during pregnancy in women whose pancreatic function is insufficient to overcome the insulin resistance associated with the pregnant state, resulting in hyperglycemia [2]. There has been a consistent rise in global morbidity [3], making GDM one of the most prevalent metabolic issues during pregnancy [4]. Despite being identified and treated during pregnancy, GDM affects both the mother’s and the fetus’s health for the rest of their lives. For women with a history of GDM, they are at an increasing risk of developing T2DM, cardiovascular disease (CVD), and chronic kidney disease (CKD). Their offspring are more likely to have metabolic disorders such as diabetes, hypertension, and obesity [5]. Large-scale epidemiological investigations have emphasized the long-term hazards [6,7].

Genome-wide association studies (GWAS), transcriptome-wide association studies (TWAS), plasma proteomics have significantly improved our knowledge regarding the genetic, genomic and proteomic architecture of GDM [3,8–14]. Elliott *et al.* discovered 13 GDM-associated loci using the largest GWAS of GDM to date, indicating both shared and different genetic architectures between GDM and type 2 diabetes [8]. Shan *et al.* nominated *NPC1* and *KIAA1191* as novel GDM risk genes with a series of popular analytic tool [15]. Although single-cell RNA sequencing (scRNA-seq) has revealed cell-type-specific transcriptional alterations in placental tissues from GDM pregnancies [16], these data alone cannot link genetic variation to gene regulation. Recent advances in single-cell expression quantitative trait locus (sc-eQTL) mapping now enable direct association of genotypes with gene expression at single cell resolution, including OneK1K [17] and TenK10K [18]. Powell *et al.* analyzed over one million immune cells from 1,000 individuals and demonstrated that the majority of eQTLs are active only in specific cell states or subtypes—a finding masked in bulk tissue analyses—thereby establishing a foundational framework for interpreting non-coding disease variants [17]. Importantly, sc-eQTL studies have shown that disease-associated SNPs often regulate gene expression exclusively in rare or context-dependent cell populations [19,20]. For instance, Chen et al. identified thousands of cell-type-restricted sc-eQTLs in human tumors, with strong enrichment near transcription start sites and dramatic variability in detection across lineages [19]. Recently, the scTWAS Atlas was launched as a comprehensive knowledgebase for single-cell transcriptome-wide association studies, integrating precomputed scTWAS results across multiple cell types and complex traits [21]. While this resource provides valuable insights into cell-type-specific genetic architecture, it currently lacks coverage of pregnancy-related conditions such as GDM and does not include specialized immune or metabolic cell states relevant to maternal-fetal physiology. Moreover, as a static repository built upon existing sc-eQTL references, it cannot accommodate newly released GWAS summary statistics—such as the large-scale FinnGen GDM data used here—for customized, up-to-date scTWAS interrogation. Therefore, a dedicated analysis leveraging state-of-the-art frameworks like OTTERS remains essential to uncover GDM-associated genes at cellular resolution. However, GDM pathogenesis extends beyond systemic immunity to the placenta, where heterogeneous cell types—including diverse trophoblasts and resident immune cells—undergo significant disease-specific transcriptional reprogramming.

**Fig 1:**
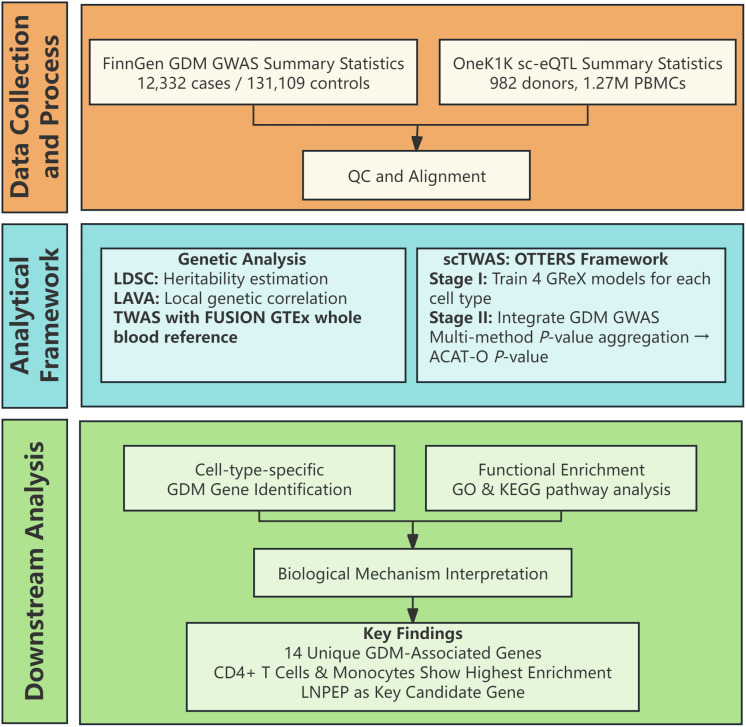
Workflow of traditional TWAS analysis using GTEx whole blood eQTL data for GDM.

Overall, existing studies have defined significant loci and genes by in- and cross-ancestry GWAS and post-GWAS analysis and cell-type specific genes by scRNA [22,23]. Current population studies primarily rely on bulk tissue data, which overlooks cellular heterogeneity and may obscure cell-type-specific genetic effects. Single-cell studies are often limited by smaller sample sizes, despite revealing stronger, more precise signals in specific cell types [21]. These drawbacks make the findings at the cellular mechanism level with small sample size difficult to use at the population level. To address this issue, leveraging the summary statistics of sc-eQTL, we mainly defined the significant genes in single-cell resolution for GDM [24]. Subsequent enrichment analysis of these genes across different cell types reveals their involvement in distinct biological pathways, providing deeper insights into the pathophysiology of GDM. We also performed a side analysis for the traditional TWAS analysis using the eQTL of whole blood from GTEx (Fig 1).

## Methods

### Data resource

We received summary statistics for GDM from the FinnGen study, which included 12,332 cases and 131,109 female controls with Finnish ancestry [8]. Following protocols used in [25–27], we performed a strict process for SNP quality control (QC): removing variants with missing effect alleles or standard errors, and retaining only SNPs present in the HapMap3 (hm3) reference panel. We maintained 1,266,324 high-quality SNPs. This improved dataset was then utilized to conduct single-cell transcriptome-wide association study (scTWAS) analysis to detect cell-type-specific genetic correlations with GDM.

Based on the OneK1K cohort, we used scRNA-seq data from about 1.27 million peripheral blood mononuclear cells (PBMCs) collected from 982 healthy donors [17]. The original dataset underwent rigorous quality control: SNPs with call rate < 95%, minor allele frequency (MAF) < 1%, or Hardy–Weinberg equilibrium *P* < 1 × 10^−6^ were excluded prior to imputation [17]. To ensure compatibility with the GDM GWAS summary statistics and enable accurate functional interpretation in the current genomic reference, we converted the OneK1K cis-eQTL mappings from GRCh37 to GRCh38 using liftOver. Following coordinate conversion, we harmonized alleles and effect directions between the eQTL and GWAS datasets. This alignment yielded 1,009,861 shared SNPs, which served as the foundation for genetically regulated expression (GReX) modeling in the OTTERS framework. We analyzed eQTL data from 12 immune cell subtypes: CD4^+^ effector memory T cells (CD4_ET_), CD4^+^ naive and central memory T cells (CD4_NC_), CD4^+^ T cells expressing SOX4 (CD4_SOX4_), CD8^+^ naive and central memory T cells (CD8_NC_), CD8^+^ T cells with expression of S100B (CD8_S100B_), CD8^+^ effector memory T cells (CD8_ET_), Memory B cells (B_Mem_), Immature and naive B cells (B_IN_), Plasma cells (Plasma), Classical monocytes (Mono_C_), Nonclassical monocytes (Mono_NC_), and Dendritic cells (DC).

### Genetic analysis for GDM

Following [3], we performed the post-GWAS analysis of GDM in two levels. At the trait level, we first applied LD Score Regression (LDSC; v1.0.1) [28] to the GDM GWAS summary statistics to estimate SNP-based heritability and assess potential confounding factors such as population stratification and cryptic relatedness. Next, we performed local heritability partitioning using Local Analysis of [co]Variant Association (LAVA; v0.1.0) [29]. To assess whether a genomic locus is enriched for heritability, we compared the observed local heritability to a null distribution generated by permuting SNP effects across the genome. Loci with permutation-based *P*-values below the Bonferroni-corrected threshold were considered statistically significant, indicating excess heritability beyond genomic background. LAVA jointly observed and latent genetic effects while accounting for Linkage Disequilibrium (LD) and polygenicity across the genome. The analysis partitioned the genome into non-overlapping LD blocks based on the 1000 Genomes Project EUR reference panel, and each locus was tested for significant enrichment of GDM heritability beyond the null expectation. Empirical P-values were derived via permutation, and significance was defined after Bonferroni correction (*P* = 0.05/ number of loci). At the molecular level, we conducted a TWAS using the FUSION framework [30]. FUSION integrates GWAS summary statistics with precomputed expression prediction models derived from GTEx whole blood to test for associations between genetically regulated gene expression and GDM risk. The analysis automatically restricts SNPs present in both the GWAS input and the expression weights, accounting for local linkage disequilibrium using a 1000 Genomes Project European reference panel. Gene-level P values were adjusted for multiple testing via the Benjamini–Hochberg procedure, and results with false discovery rate (FDR) < 0.05 were considered significant. Of note, LAVA local heritability estimates are not constrained to sum to the genome-wide LDSC heritability and may appear larger due to local LD structures.

### scTWAS analysis

We performed scTWAS using the OTTERS framework [24], which integrates multiple expression imputation models to enhance robustness in gene–trait association testing. Critically, we independently implemented Stage I of OTTERS—the computationally intensive step of training gene-level GReX imputation models across all autosomal genes. Specifically, for each of the 12 immune cell types profiled in the OneK1K sc-eQTL resource, we trained four distinct GReX models per gene using lassosum, SDPR, PRS-CS, and a frequentist P + T baseline, as implemented in OTTERS. These models were derived from summary-level cis-eQTL statistics provided by the OneK1K consortium, combined with an ancestry-matched LD reference panel constructed from 503 European-ancestry individuals in the 1000 Genomes Project (Phase 3).

To ensure biological relevance and computational consistency, gene annotations were obtained from GENCODE v41 (https://www.gencodegenes.org/), and cis-regions were defined as ±1 Mb around each gene’s transcription start (GeneStart) and end (GeneEnd) sites. All model training was performed on high-performance computing clusters, requiring substantial computational resources due to the scale of genome-wide modeling across multiple cell types and methods. The resulting eQTL weight files—comprising over 200,000 gene–cell–method combinations—have been made publicly available via Figshare (https://doi.org/10.6084/m9.figshare.30632813) to support future scTWAS applications in pregnancy-related or immune-mediated traits.

In Stage II, we applied these pre-trained models to the GDM GWAS summary statistics to impute GReX and compute gene-level association Z-scores for each method and cell type. Finally, we aggregated the four method-specific TWAS *P* values per gene using the ACAT-O omnibus test to produce a single [34], robust association statistic that leverages complementary strengths of diverse imputation strategies.

Different multiple testing correction strategies were employed based on the specific goals of each analysis stage. For the genome-wide gene discovery phases (scTWAS and bulk TWAS), we controlled for multiple comparisons using the FDR to maximize statistical power and minimize false negatives in this exploratory setting. In contrast, for the LAVA, we utilized Bayes Factors (BF). The Bayesian framework was chosen for LAVA as it provides a direct measure of evidence strength for shared genetic architecture without relying on asymptotic *p*-value assumptions, offering a more robust assessment for focused regional analyses. For each gene, OTTERS requires at least one significant cis-eQTL (FDR < 0.05) in the reference data to build GReX models; thus, only genes with detectable genetic regulation were included.

### Enrichment analysis

We used *clusterProfiler* package (v4.10.1) to conduct functional enrichment analysis on gene sets resulting from ACAT results [31–33]. This program allows for over-representation analysis (ORA) of GO and KEGG pathways, as well as comparative display of enriched biological topics across gene clusters. We utilized clusterProfiler’s enrichGO and enrichKEGG algorithms with default parameters to discover significantly enriched phrases, which were then visualized using the built-in dot and bubble plots. Gene annotations were based on human genome databases, which aligned with the package’s recommended process for human data.

### Ethics statement

This study used publicly available, de-identified summary data. The FinnGen GDM GWAS data were collected with ethics approval from the Coordinating Ethics Committee of the Hospital District of Helsinki and Uusimaa (HUS/990/2017) and participant informed consent. The OneK1K sc-eQTL data were collected with ethics approval from the Tasmania Health and Medical Human Research Ethics Committee (H0012902) and participant written informed consent. This secondary analysis of anonymized data was exempt from further review by the Ethics Committee of Nanjing Medical University.

## Results

### Post-GWAS analysis for GDM

**Fig 2:**
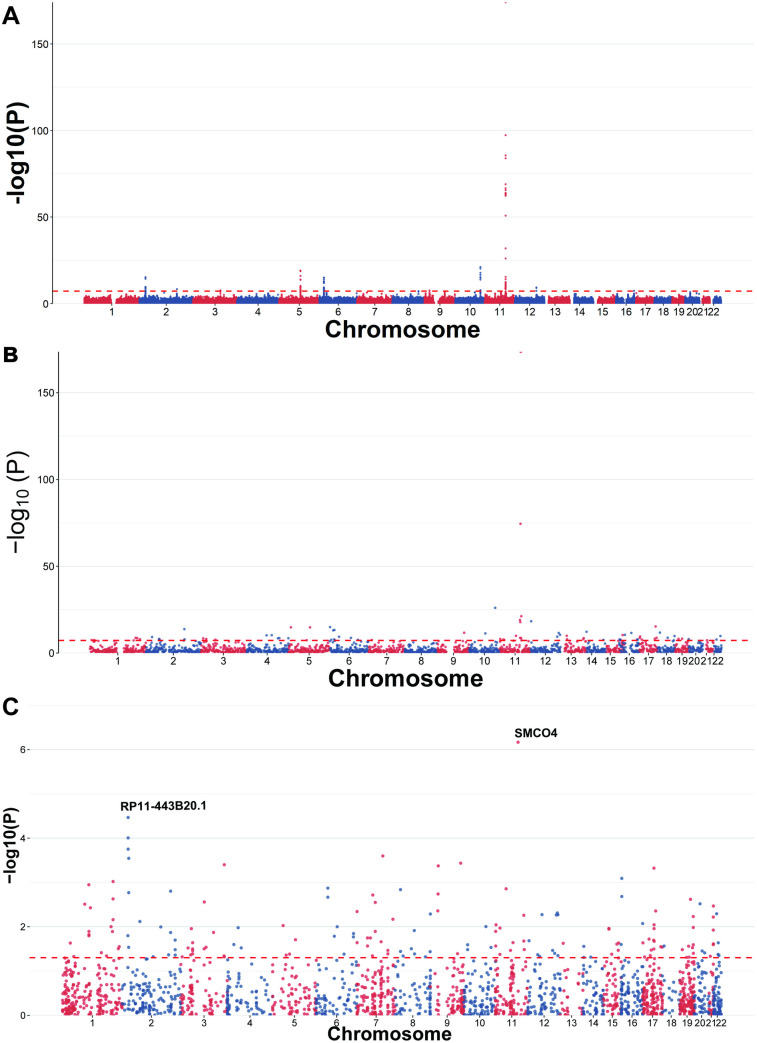
Summary for the post-GWAS analysis of GDM. (A) Manhattan plot for summary statistics; (B) Manhattan plot for local heritability; (C) Manhattan plot for FUSION.

Based on LDSC, the observed SNP-based heritability of GDM was 0.0352 (standard error [SE] = 0.0052, *P* < 0.001; Fig 2A). To further dissect the genomic architecture, LAVA identified 828 genomic regions significantly enriched for GDM heritability after Bonferroni correction (α = 0.05/ number of loci), with the strongest signals on chromosomes 11 (e.g., chr11: 92.5–93.8 Mb, local h² = 0.0236, *P* = 2.77 × 10^−174^) and 2 (e.g., chr2: 168.4–169.3 Mb, local h² = 0.00367, *P* = 1.73 × 10^−14^) (Fig 2B and Table 1). Using TWAS with GTEx whole blood gene expression models, we identified two genes showing nominal associations with GDM: *SMCO4* (TWAS. Z = 4.97, TWAS. *P* = 6.82 × 10^−7^) and *RP11-443B20.1* (TWAS. Z = 4.14, TWAS. *P* = 3.42 × 10^−5^). However, neither gene remained significant after FDR correction for multiple testing (q > 0.05)(Fig 2C). The top 20 nominal associations are presented in Table 2.

**Table 1:** Top 10 genomic loci contributing to GDM heritability.

Block Number	Genomic Block Range	Gene Number	Local h^2^	*P* Value	FDR
1633	92,476,003-93,843,475	47	0.023619	2.77 × 10^-174^	5.53 × 10^-171^
1632	91,233,894−92,476,002	15	0.011586	3.16 × 10^-75^	3.15 × 10^-72^
1531	113,829,145−115,085,454	45	0.00643	8.76 × 10^-27^	5.83 × 10^-24^
1635	94,651,370−95,594,046	28	0.005135	6.10 × 10^-22^	3.05 × 10^-19^
1629	88,626,120−89,476,570	5	0.003471	9.11 × 10^-20^	3.64 × 10^-17^
1676	3,761,949−4,858,477	45	0.005301	4.55 × 10^-19^	1.51 × 10^-16^
1631	90,466,765−91,233,893	10	0.003235	1.45 × 10^-18^	4.13 × 10^-16^
2142	71,249,450−72,498,979	17	0.005061	4.74 × 10^-16^	1.18 × 10^-13^
898	1,380,445−2,746,297	28	0.004797	1.08 × 10^-15^	2.41 × 10^-13^
759	10,548,918−11,850,738	12	0.004504	1.48 × 10^-15^	2.86 × 10^-13^

1^st^ column: block number; 2^nd^ column: genomic block range; 3^rd^ column: number of genes within the block; 4^th^ column: local SNP-based heritability estimate; 5^th^ column: *P* value; 6^th^ column: FDR-adjusted *P* value.

**Table 2:** Top 20 nominal associations from bulk TWAS using GTEx whole blood eQTL data.

Gene	CHR	Gene Range	Number of cis-SNP	*P* Value	FDR
*AC002467.7*	7	107,383,262−107,385,026	428	2.52 × 10^−4^	9.36 × 10^−2^
*PPP6C*	9	127,908,852−127,952,218	321	3.66 × 10^−4^	9.36 × 10^−2^
*ABCC5*	3	183,637,722−183,735,803	526	3.98 × 10^−4^	9.36 × 10^−2^
*VCP*	9	35,056,061−35,073,246	340	4.22 × 10^−4^	9.36 × 10^−2^
*SNX11*	17	46,180,719−46,200,436	450	4.75 × 10^−4^	9.48 × 10^−2^
*MMP25*	16	3,096,682−3,110,727	347	8.09 × 10^−4^	1.47 × 10^−1^
*RP3-434O14.8*	1	209,960,324−209,961,038	506	9.53 × 10^−4^	1.59 × 10^−1^
*PSRC1*	1	109,822,178−109,825,808	430	1.12 × 10^−3^	1.73 × 10^−1^
*CENPQ*	6	49,431,091−49,460,820	349	1.34 × 10^−3^	1.81 × 10^−1^
*C8orf58*	8	22,457,114−22,461,663	513	1.45 × 10^−3^	1.81 × 10^−1^
*NUDT2*	9	34,329,504−34,343,709	337	1.82 × 10^−3^	1.91 × 10^−1^
*GS1-124K5.12*	7	66,010,634−66,057,373	250	1.92 × 10^−3^	1.92 × 10^−1^
*RP11-473M20.7*	16	3,101,992−3,109,371	347	2.08 × 10^−3^	1.95 × 10^−1^
*MUT*	6	49,398,073−49,430,904	353	2.15 × 10^−3^	1.95 × 10^−1^
*IRF6*	1	209,959,036-209,979,465	518	2.36 × 10^−3^	2.00 × 10^−1^
*XRCC1*	19	44,047,192−44,084,625	382	2.40 × 10^−3^	2.00 × 10^−1^
*SENP7*	3	101,043,049-101,232,085	461	2.76 × 10^−3^	2.16 × 10^−1^
*POMZP3*	7	76,239,303−76,256,578	155	2.82 × 10^−3^	2.16 × 10^−1^
*PYGB*	20	25,228,705−25,278,650	422	3.03 × 10^−3^	2.20 × 10^−1^
*EVI5*	1	92,974,253−93,257,961	344	3.09 × 10^−3^	2.20 × 10^−1^

1^st^ column: gene symbol; 2^nd^ column: chromosome; 3^rd^ column: Genomic coordinates; 4^th^ column: the number of cis-SNP; 5^th^ column: *P* value; 6^th^ column: FDR-adjusted *P* value.

### sc-eQTL

**Fig 3:**
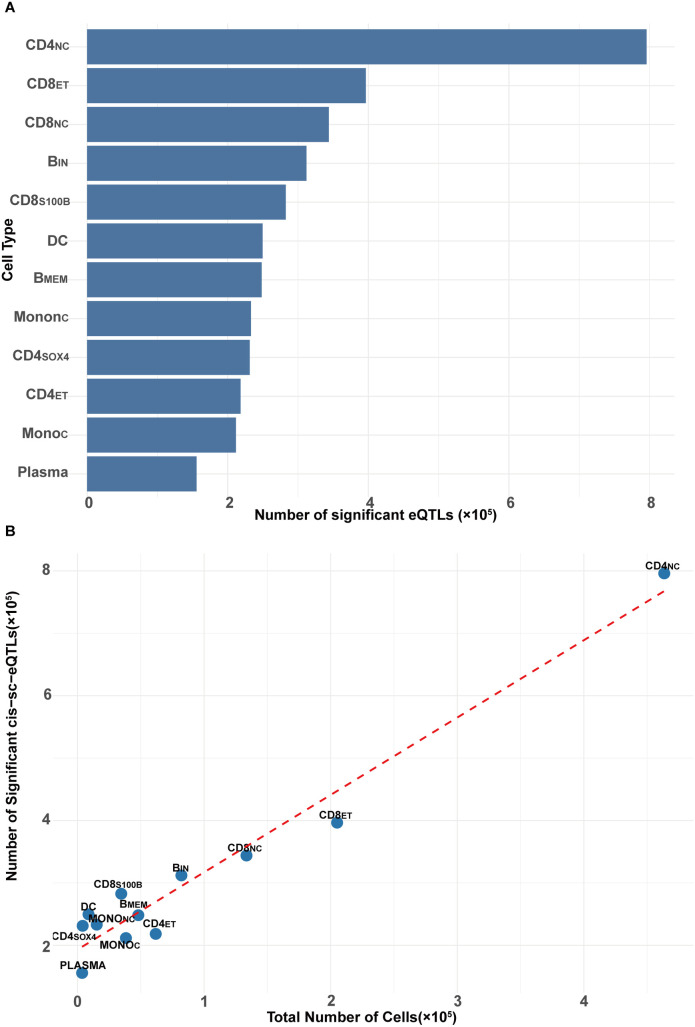
(A) Bar plot indicates the significant eQTL number across each cell type. (B) Correlation between the number of cells per type and the total count of significant eQTLs.

We quantified the number of significant cis-sc-eQTLs (FDR < 0.05) across 12 immune cell types in the OneK1K cohort. CD4_NC_ exhibited the highest regulatory complexity, with 795,929 significant eQTLs—more than twice that of other major lineages. CD8_ET_ and CD8_NC_ followed, with 396,523 and 343,885 eQTLs, respectively. In contrast, plasma cells showed the lowest number (155,713), while B cells, dendritic cells, and monocyte subsets displayed intermediate levels (Fig 3). This pronounced heterogeneity underscores the cell-type-specific nature of genetic regulation and positions CD4^+^ T cells as a key cellular context for downstream GDM association analysis. However, as shown in the linear correlation between cell abundance and eQTL counts (Fig 3B), sample size is a major driver of these differences (S2 Table). Therefore, the prominence of CD4_NC_ cells reflects both their high population frequency and substantial regulatory activity, providing the robust statistical power necessary to reliably detect disease-associated signals.

**Fig 4:**
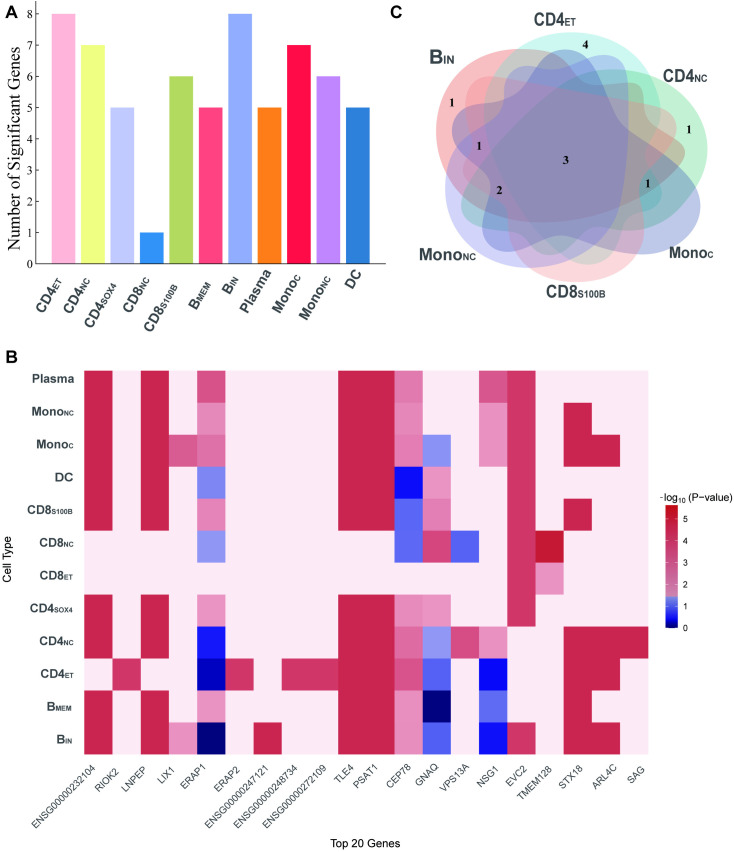
Summary for the sc-eQTL analysis. (A) Bar plot indicates the number of significant genes across each cell type. (B) Heatmaps show the P value for the top 20 genes. (C) Venn diagram representing six cell populations.

We performed single-cell transcriptome-wide association analysis (sc-TWAS) by integrating OTTERS with ACAT across 12 immune cell types from the OneK1K cohort. After FDR correction (q < 0.05), a total of 63 significant gene–cell type associations were detected; however, these corresponded to only 14 unique genes after removing duplicates. The number of significant associations varied by cell type, with the highest counts in CD4_ET_ and B_IN_ (8 each), followed by CD4_NC_ and Mono_C_ (7 each), while CD8-ET showed no significant associations (Fig 4A). Among the top 20 genes with the smallest ACAT p-values across all cell types, several displayed cell-type-specific patterns (Fig 4B). For instance, *RIOK2* and *ERAP1* were significant in nearly all 12 cell types (ACAT. *P* = 2.17 × 10^−5^). A Venn diagram of the 14 unique significant genes across six cell populations (CD4_ET_, CD4_NC_, CD8_S100B_, B_IN_, Mono_C_, and Mono_NC_) revealed that most genes were specific to one or two cell types, with minimal sharing between T-cell and myeloid subsets (Fig 4C). Combined with the higher burden of significant genes in CD4 + T cells and monocytes (Fig 4A), these findings suggest that these immune lineages may play distinct and important roles in GDM pathogenesis. To distinguish true causal genes from potential false positives driven by LD, we performed statistical colocalization analysis (COLOC) for all 14 unique genes across the 12 cell types (S1 Table). Remarkably, among all candidates, only *LNPEP* demonstrated strong evidence of colocalization (PP.H4 > 0.7) in MONO_c_, indicating that the GWAS and sc-eQTL signals share a single underlying causal variant. In contrast, other top hits including *ERAP1*, *ERAP2*, and *RIOK2* did not show strong colocalization signals (PP.H4 < 0.5) in the analyzed immune subsets; many exhibited elevated PP.H3 probabilities, suggesting that their TWAS associations may arise from distinct causal variants in LD or are specific to tissues not profiled in this study. This rigorous validation step refines our candidate list, establishing LNPEP as a high-confidence colocalization-prioritized candidate gene for GDM, with its strongest support in monocytes.

### Enrichment analysis

**Fig 5:**
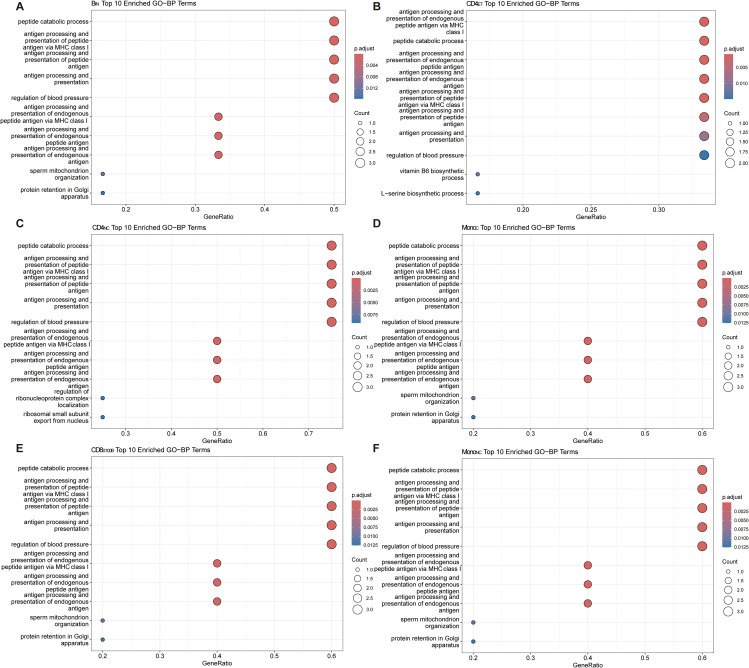
Gene Ontology enrichment of GDM-associated genes in B_IN_, CD4_ET_, CD4_NC_, Mono_C_, CD8_S100B_, and Mono_NC_ (FDR < 0.05).

We performed GO enrichment analysis on the 14 unique GDM-associated genes identified by sc-TWAS across six immune cell types (CD4_ET_, CD4_NC_, CD8_S100B_, B_IN_, Mono_C_, and Mono_NC_) (Fig 5C). The top enriched terms consistently included “antigen processing and presentation of peptide antigen via MHC class I” (FDR < 10^−5^), “peptide catabolic process” (FDR < 10^−5^), and related peptidase activities. Functional enrichment highlighted divergent roles: BIN cells were enriched for metalloaminopeptidase activity (*P* = 4.92 × 10^−8^) and MHC class I-mediated antigen presentation (*P* = 1.27 × 10^−7^). CD4ET cells shared peptidase functions but were specifically linked to endogenous antigen presentation (*P* = 2.13 × 10^−5^) and spondylitis (*P* = 2.55 × 10^−4^).

## Discussion

In this study, we leveraged sc-TWAS to dissect the cell-type-specific genetic architecture of GDM. Compared to conventional bulk-tissue TWAS, such as our FUSION-based analysis using GTEx whole blood data, which identified two genes showing nominal associations (*SMCO4* and *RP11-443B20.1*) but neither survived FDR correction, our sc-TWAS approach integrating OTTERS and ACAT across 12 immune cell types revealed 14 unique GDM-associated genes, many of which showed cell-type-specific association patterns. Notably, scTWAS identified more GDM-associated genes than bulk TWAS using GTEx whole blood, although this comparison is limited by differences in methods and reference data [21,34].

A key finding of our integrated analysis is the identification of LNPEP as a high-confidence candidate causal gene in monocytes, although functional validation in pregnancy-relevant contexts is needed. While multiple genes showed significant TWAS associations, colocalization analysis confirmed that only *LNPEP* shares a single causal variant with GDM susceptibility (PP.H4 > 0.7), specifically in classical monocytes. *LNPEP* is a multifunctional aminopeptidase critical for degrading oxytocin and vasopressin during pregnancy [35], but it also plays an essential role in trimming peptides for MHC class I antigen presentation [36]. Monocytes are pivotal drivers of the low-grade inflammation and insulin resistance characteristic of GDM [37]. Our finding suggests that genetic variants regulating *LNPEP* expression in monocytes may disrupt local peptide hormone homeostasis or alter antigen processing capabilities, thereby promoting a pro-inflammatory state that exacerbates systemic insulin resistance. This positions LNPEP as a strong candidate for further mechanistic investigation, potentially acting through the monocyte-macrophage axis, warranting urgent functional validation.

Interestingly, *SMCO4* and *RP11-443B20.1*, which were significant in our bulk-tissue TWAS, did not reach significance in this single-cell analysis. This discrepancy likely reflects that their bulk signals arise from aggregated effects across multiple cell types or are driven by tissues not captured in our peripheral blood reference panel. While our scTWAS successfully identified immune-specific drivers, the absence of these two genes at single-cell resolution suggests their functional impact on GDM may be mediated by non-immune compartments or subtle, coordinated changes below the detection threshold of current immune atlases. Future studies in metabolically active tissues will be needed to resolve their precise cellular context.

In contrast, other prominent candidates such as *ERAP1*, *ERAP2*, and *RIOK2*, while showing pervasive TWAS signals, did not demonstrate strong colocalization in our immune cell dataset. *ERAP1* and *ERAP2* are well-established regulators of MHC class I antigen presentation implicated in autoimmune diseases [38]. The lack of colocalization in peripheral blood immune cells suggests two possibilities: (i) their causal effects on GDM may be mediated through other tissues not captured here (e.g., pancreatic beta cells, liver, or placenta); or (ii) their TWAS signals may be partially confounded by LD with distinct causal variants. Similarly, *RIOK2* showed broad associations but lacked colocalization support. These results highlight the importance of colocalization in refining TWAS hits: while *ERAP1/2* and *RIOK2* remain biologically plausible, LNPEP emerges as the most robust locus supported by both association and shared causal variation in the studied cell types.

Despite these differences, the functional convergence points toward immune dysregulation as a central theme. The enrichment of ‘antigen processing and presentation’ pathways, driven largely by the *ERAP* family signals, aligns with the hypothesis that altered maternal immune tolerance contributes to GDM [38]. Whether mediated directly by *LNPEP* in monocytes or indirectly through *ERAP*-related pathways in other tissues, our data suggest that aberrant peptide handling creates a pro-inflammatory environment exacerbating insulin resistance.

### Limitation

Despite these insights, several limitations warrant consideration. First, our analysis relies exclusively on peripheral blood sc-eQTL data due to the unavailability of large-scale placental references. Consequently, we could not assess disease mechanisms in key placental populations. The identified immune-cell-specific associations thus represent only part of the overall tissue-specific architecture underlying GDM. Second, the GDM GWAS summary statistics were based exclusively on individuals of Finnish ancestry, which limits the generalizability of our results to other populations. Third, while sc-TWAS has more resolution than bulk-tissue techniques, it is still an observational method that infers gene-trait relationships by statistical integration; causation cannot be shown without functional confirmation. Finally, current sc-eQTL resources are still constrained by sample size, which may reduce power to detect associations in rare or low-abundance cell subsets. Despite high allele frequency concordance between FinnGen and 1000 Genomes EUR, residual LD differences may still affect GReX imputation and should be interpreted with caution.

## Supporting information

S1 Table Colocalization results for GDM-associated genes across 12 immune cell types. (XLSX)

S2 Table Summary of cis-sc-eQTL counts and cell abundance across 12 immune cell types. (XLSX)
